# *Diaporthe foeniculina* and *D. eres*, in addition to *D. ampelina*, may cause Phomopsis cane and leaf spot disease in grapevine

**DOI:** 10.3389/fpls.2024.1446663

**Published:** 2024-09-02

**Authors:** Giorgia Fedele, Josep Armengol, Tito Caffi, Luca Languasco, Nedeljko Latinovic, Jelena Latinovic, Maela León, Guido Marchi, Laura Mugnai, Vittorio Rossi

**Affiliations:** ^1^ Department of Sustainable Crop Production (DI.PRO.VE.S.), Università Cattolica del Sacro Cuore, Piacenza, Italy; ^2^ Instituto Agroforestal Mediterráneo, Universitat Politècnica de València, Valencia, Spain; ^3^ Biotechnical Faculty, University of Montenegro, Podgorica, Montenegro; ^4^ Dipartimento di Scienze e Tecnologie Agrarie, Alimentari, Ambientali e Forestali (DAGRI), Sezione di Patologia Vegetale ed Entomologia, Università di Firenze, Florence, Italy

**Keywords:** *Diaporthe neotheicola*, fungal isolation, molecular identification, phylogenetic analysis, temperature-dependent growth, pathogenicity

## Abstract

Phomopsis cane and leaf spot (PCLS) disease, affecting grapevines (*Vitis vinifera* and *Vitis* spp.), has been historically associated with *Diaporthe ampelina*. Typical disease symptoms, comprising bleaching and black pycnidia, have also been associated with other *Diaporthe* spp. In this study, we conducted a molecular identification of the *Diaporthe* isolates isolated from grapevine canes from different geographic areas of southern Europe showing PCLS symptoms. Then, we investigated their morphological characteristics (including mycelium growth and production of pycnidia and alpha and beta conidia) in response to temperature. Finally, we artificially inoculated grapevine shoots and leaves with a subset of these isolates. Based on our results, PCLS etiology should be reconsidered. Though *D. ampelina* was the most crucial causal agent of PCLS, *D. eres* and *D. foeniculina* were also pathogenic when inoculated on green shoots and leaves of grapevines. However, *D. rudis* was not pathogenic. Compared to *D. ampelina*, *D. eres* and *D. foeniculina* produced both pycnidia and alpha conidia at lower temperatures. Thus, the range of environmental conditions favorable for PCLS development needs to be widened. Our findings warrant further validation by future studies aimed at ascertaining whether the differences in temperature requirements among species are also valid for conidia-mediated infection since it could have substantial practical implications in PCLS management.

## 1 Introduction

Phomopsis cane and leaf spot (PCLS) affects grapevines (*Vitis vinifera* and *Vitis* spp.) wherever grapes are grown (Pearson and Goheen, 1994), even though it is more severe in grape-growing regions characterized by a humid temperate climate throughout the growing season. Previous studies have reported crop losses of up to 30% or sometimes even 50% due to PCLS (Pine, 1958; Berrysmith, 1962; Pscheidt and Pearson, 1989; Erincik et al., 2003). This disease results in the breaking off of shoots at the base, stunted growth, reduced bunch set, and berry rot (Pine, 1958, 1959; Pscheidt and Pearson, 1989; Pearson and Goheen, 1994).

PCLS can affect all green parts of the grapevine, and the symptoms primarily emerge early in the season after budburst but before full canopy development makes the basal internodes barely visible. The shoots, especially the basal part, of the affected plants exhibit brown to black necrotic irregular-shaped lesions, often with longitudinal cracks. In the affected leaves, PCLS manifests as small, pale green to yellow spots with necrotic centers (Pearson and Goheen, 1994). The symptoms in clusters usually emerge when the fruit begins to ripen, with rachises becoming necrotic and berries rotting or falling to the ground (Erincik et al., 2002). In winter, the affected canes exhibit bleached white areas speckled with small black spots (the pycnidia). Black cracks are also evident in case of severe symptoms on the canes.

PCLS is historically associated with *Diaporthe ampelina* (syn. *Phomopsis viticola*) (Pearson and Goheen, 1994). In a previous study aimed at developing a mathematical model to simulate PCLS epidemics (González-Domínguez et al., 2021), we isolated *Diaporthe* spp. from grapevine canes showing typical disease symptoms, with bleaching and black pycnidia. The morphologies of some of these isolates resembled that of *D. ampelina*, while others had distinctive morphology, suggesting possible differences in their taxonomy.

*Diaporthe* spp. other than *D. ampelina* have previously been found in grapevine wood; however, none have been strongly associated with typical PCLS symptoms. For instance, *D. perjuncta* has been associated with cane bleaching (comprising bleached canes with black fruiting bodies) (Merrin et al., 1995), but artificial inoculation studies have shown that this species is an endophyte, rather than a pathogen of grapevine (Mostert et al., 2001; Rawnsley et al., 2004). *D. kyushuensis* (a teleomorph of *P. vitimegaspora*) is considered the causal agent of grapevine swelling arm disease (Kuo and Leu, 1998; Kajitani and Kanematsu, 2000). *P. amygdali* has been isolated from grapevines grown in the vineyards of South Africa and was found to cause dark-brown lesions similar to those caused by *D. ampelina* when wound-inoculated on green shoots (Mostert et al., 2001). Guarnaccia et al. (2018) isolated nine *Diaporthe* spp. (namely, *D. ambigua*, *D. ampelina*, *D. baccae*, *D. bohemiae*, *D. celeris*, *D. eres*, *D. hispaniae*, *D. hungariae*, and *D. rudis*) from both asymptomatic and symptomatic parts (canes, cordons, and trunks) of grapevines from the vineyards in seven European countries (Croatia, Czech Republic, France, Hungary, Italy, Spain, and the UK) and Israel. They reported that the symptoms included cane and leaf spot, cane bleaching, vascular browning, and/or sectorial necrosis in the wood. In addition, all the isolates, except for *D. bohemiae*, caused necrotic lesions on inoculated grapevine shoots. However, the specific disease symptoms caused by each isolate were not reported.

*Diaporthe* spp. have also been associated with grapevine cankers (Úrbez-Torres et al., 2009, 2012, 2013; Baumgartner et al., 2013). *D. ampelina* can infect and colonize mature grapevine wood and develop cankers beyond the point of inoculation as demonstrated by several artificial inoculation studies (Reddick, 1909, 1914; Coleman, 1928; Chamberlain et al., 1964). Baumgartner et al. (2013) observed a frequent co-occurrence of the foliar symptoms of PCLS and wood cankers. Notably, in addition to *P. viticola*, *P. fukushii* and *D. eres* have also been isolated from such cankers. Furthermore, *Phomopsis theicola*, the anamorph of *D. neotheicola*, reportedly causes the Esca disease in grapevines; however, its pathogenicity has not yet been explored (White et al., 2011). In the current study, we referred to *D. neotheicola* by its synonym *D. foeniculina* (Udayanga et al., 2014). A previous study proposed the inclusion of Diaporthe dieback into the grapevine trunk disease (GTD) complex after providing strong evidence about the role of *D. ampelina* as a canker-causing organism (Úrbez-Torres et al., 2013). Its symptoms include a general vine decline, shoot dieback, and dead spurs with perennial cankers and vascular discoloration, similar to the symptoms of GTDs Botryosphaeria dieback and Eutypa dieback caused by Botryosphaeriaceae spp. and *Eutypa lata*, respectively (Úrbez-Torres et al., 2013; Baumgartner et al., 2013). *Diaporthe eres*, *D. ambigua*, and other species have also been isolated from grapevine cankers in California (Lawrence et al., 2015). Some of these species are considered saprophytes on grapevine wood (Úrbez-Torres et al., 2013), while others are considered weak to moderate pathogens causing wood cankers (Kaliterna et al., 2012; Baumgartner et al., 2013). *Diaporthe* spp. have also been found to colonize the internal wood of symptomatic (internal vascular necrosis) and asymptomatic plants in grapevine nurseries (Carbone et al., 2022).

In the present study, we conducted a molecular identification of representative fungal isolates obtained from grapevines showing PCLS symptoms in Mediterranean, European countries. Then, we analyzed the morphological characteristics of the isolates (mycelium growth and production of pycnidia and alpha and beta conidia) at varying temperatures. Finally, we artificially inoculated the grapevine shoots and leaves with each isolate to assess its potential role in PCLS.

## 2 Materials and methods

### 2.1 Collection of fungal isolates

During 2016, the vineyards in Podgorica, Montenegro, were surveyed. The cane samples showing bleaching with longitudinal lesions, which are typical PCLS symptoms, were collected from cultivar Vranac, the most cropped variety in that country. The samples were surface sterilized with 75% ethanol for 10 s, followed by 2% sodium hypochlorite solution for 2 min. The bark was removed from the samples to reveal internal necrosis and/or symptoms of browning. Small tissue pieces were extracted from the margin between necrotic/discolored regions and apparently healthy tissues using a sterile scalpel. From cane samples with external PCLS symptoms but without any internal necrosis, pieces of apparently healthy wood were taken at random after removing the bark. All the tissue pieces were plated onto potato dextrose agar (PDA) (Biolife Italiana, Milan, Italy) supplemented with 100 mg/L streptomycin sulfate (Merck Life Science, Milan, Italy).

The culture plates were incubated at 25°C in the dark until fungal colonies emerged. The colonies with *Diaporthe* spp. morphology were subcultured and purified by transferring the hyphal tips to fresh PDA plates (Baumgartner et al., 2013). These plates were incubated at 25°C under white light in 12-h light/12-h dark cycles for 4 weeks to stimulate the production of pycnidia and alpha and/or beta conidia (Baumgartner et al., 2013; Úrbez-Torres et al., 2013; Guarnaccia et al., 2018). Isolates with confirmed *Diaporthe* spp. characteristics were stored on 1.5% water agar (WA) (Biolife Italiana, Milan, Italy) at 4°C and deposited in the fungal culture collection of the Department of Sustainable Crop Production, Università Cattolica del Sacro Cuore, Piacenza, Italy.

In addition, isolates previously collected in different viticultural areas of Italy and Kosovo from canes showing typical PCLS symptoms and maintained at the culture collection of the University of Florence (Italy) as well as two isolates also obtained from PCLS symptoms in Spain were included in this study (**Table 1**).

**Table 1:** Diaporthe isolates isolated from *Vitis vinifera* and their use in experiments related to the analysis of morphological traits (1 for mycelium growth and 2 for production of pycnidia and conidia) and pathogenicity (3 and 4 for inoculation with mycelia and conidia, respectively).

				Experiments			
Fungal species	Country of origin	Isolate code	Disease and organ	1	2	3	4
*Diaporthe ampelina*	Italy (North)	*Dam*_IT1	PCLS, canes with pycnidia	X	X	X	X
*D. ampelina*	Italy (central)	*Dam*_IT2	PCLS, canes with pycnidia	X	X	X	X
*D. ampelina*	Italy (South)	*Dam*_IT3	PCLS, canes with pycnidia	X	X	X	X
*D. ampelina*	Montenegro (South)	*Dam*_MNE1	PCLS	X	X	X	X
*D. ampelina*	Montenegro (South)	*Dam*_MNE2	PCLS, canes with pycnidia	X	X		
*D. ampelina*	Montenegro (South)	*Dam*_MNE3	PCLS	X	X		
*D. ampelina*	Montenegro (South)	*Dam*_MNE4	PCLS	X	X		
*D. ampelina*	Montenegro (South)	*Dam*_MNE5	PCLS	X	X		
*D. ampelina*	Spain (East)	*Dam_*SP1	PCLS		X	X	X
*D. ampelina*	Spain (East)	*Dam_*SP2	PCLS		X	X	X
*Diaporthe eres*	Italy	*Der*_IT1	PCLS, canes with pycnidia	X	X	X	X
*D. eres*	Kosovo	*Der*_RKS1	PCLS	X	X	X	X
*Diaporthe foeniculina*	Montenegro (South)	*Dfo*_MNE1	PCLS	X	X	X	X
*Diaporthe rudis*	Italy (central)	*Dru*_IT2	PCLS, canes with pycnidia	X	X	X	
*D. rudis*	Italy (North)	*Dru*_IT1	PCLS, canes with pycnidia	X	X	X	
*D. rudis*	Italy	*Dru*_IT3	PCLS, canes with pycnidia	X	X		

X: Performed experiment.

### 2.2 Molecular identification of *Diaporthe* spp. isolates

#### 2.2.1 DNA extraction, polymerase chain reaction amplification, and sequencing

Genomic DNA was extracted from the mycelia of pure fungal cultures as previously described by Leon et al. (2020). The internal transcribed spacer (ITS) region, part of the beta-tubulin gene region (*tub*), partial translation elongation factor 1-alpha (*tef1-α*) gene, histone H3 (*his*) gene, and calmodulin (*cal*) gene were amplified and sequenced using primers pairs included in **Supplementary Table S1**. All PCR amplifications, with a final volume of 20 μL and primer concentration of 0.3 μM, were performed using Speedy Supreme NZYTaq 2× Green Master Mix (NZYtech™, Lisbon, Portugal), according to the manufacturer’s instructions, on a Peltier Thermal Cycler-200 (MJ Research). The thermal cycle comprised an initial step of incubation at 95°C for 5 min, followed by 35 cycles of denaturation at 94°C for 2 s, annealing (at varying temperatures for different targets) for 5 s, and elongation at 72°C for 5 s. A final extension was performed at 72°C for 2 min. The annealing temperatures were 55°C for ITS, *tef1-α*, and *tub*, and 58°C for *cal* and *his*. The PCR products were analyzed using 1.2% agarose gel electrophoresis and were sequenced at IBMCP-UPV (Valencia, Spain). Each consensus sequence was assembled using Sequencher 5.0 (Gene Codes Corp., Ann Arbor, Michigan).

#### 2.2.2 Phylogenetic analyses

A primary identification of fungi was done using the Nucleotide BLAST program on the NCBI website (https://blast.ncbi.nlm.nih.gov/), and sequences of the closely related species were retrieved from GenBank. The sequences of the five loci (ITS, *tef1-α*, *tub*, *cal*, and *his*) obtained in the current study were aligned with the corresponding sequences retrieved from Genbank (**Table 2**) using the ClustalW algorithm (Thompson et al., 1994) in the MEGA11 software package (Tamura et al., 2021). The alignments were examined and corrected manually.

**Table 2:** GenBank accession numbers of sequences used for phylogenetic analyses.

Species	Isolate	GenBank accession numbers				
		ITS	*tub2*	*his3*	*tef1*	*cal*
*Diaporthe acaciigena*	CBS 129521; CPC 17622 ^T^	KC343005	KC343973	KC343489	KC343731	KC343247
*D. ambigua*	CBS 187.87	KC343015	KC343983	KC343499	KC343741	KC343257
	CBS 114015; STE-U 2657; CPC 2657 ^T^	KC343010	KC343978	KC343494	KC343736	KC343252
*D. ampelina*	CBS 111888; ATCC 48153; STE-U 2673; CPC 2673	KC343016	KC343984	KC343500	KC343742	KC343258
	CBS 114016; STE-U 2660; CPC 2660; PV F98-1 ^T^	AF230751	JX275452	–	GQ250351	JX197443
	Dam_IT2; D12	**PP803044**	**PP786212**	**PP803728**	**PP803708**	**PP803690**
	Dam_IT1; D2	**PP803040**	**PP786209**	**PP803724**	**PP803704**	**PP803687**
	Dam_IT3; D3	**PP803042**	**PP786210**	**PP803726**	**PP803706**	**PP803688**
	Dam_MNE2; PHO1	**PP803051**	**PP786219**	**PP803735**	**PP803715**	**PP803695**
	Dam_MNE3; PHO3	**PP803053**	**PP786221**	**PP803737**	**PP803717**	**PP803697**
	Dam_MNE4; PHO4	**PP803054**	**PP786222**	**PP803738**	**PP803718**	**PP803698**
	Dam_MNE1; PHO5	**PP803055**	**PP786223**	**PP803739**	**PP803719**	**PP803699**
	Dma_MNE5; PHO7	**PP803057**	**PP786225**	**PP803741**	**PP803721**	**PP803701**
	Dma_SP1; PV4	**PP803058**	**PP786226**	**PP803742**	**PP803722**	**PP803702**
	Dma_SP2; PV7	**PP803059**	**PP786227**	**PP803743**	**PP803723**	**PP803703**
*D. amygdali*	CBS 126679 ^T^	KC343022	KC343990	KC343506	KC343748	KC343264
*D. australafricana*	CBS 111886; STE-U 2676; CPC 2676 ^T^	KC343038	KC344006	KC343522	KC343764	KC343280
*D. celeris*	CBS 143349; CPC 28262 ^T^	MG281017	MG281190	MG281363	MG281538	MG281712
	CBS 143350; CPC 28266	MG281018	MG281191	MG281364	MG281539	MG281713
*D. eres*	CBS 138594; AR5193 ^T^	KJ210529	KJ420799	KJ420850	KJ210550	KJ434999
	CPC 28423; PVFi-M149	KT369109	KT369113	MG281379	KT369111	MG281728
	Der_RKS1; D10	**PP803043**	**PP786211**	**PP803727**	**PP803707**	**PP803689**
	Der_IT1; D22	**PP803048**	**PP786216**	**PP803732**	**PP803712**	**PP803693**
*D. foeniculina*	CBS 187.27 ^T^	KC343107	KC344075	KC343591	KC343833	KC343349
	CBS 111553 ^T^	KC343101	KC344069	KC343585	KC343827	KC343343
*D. hispaniae*	CBS 143351; CPC 30321 ^T^	MG281123	MG281296	MG281471	MG281644	MG281820
	CBS 143352; CPC 30323	MG281124	MG281297	MG281472	MG281645	MG281821
*D. impulsa*	CBS 114434; UPSC 3052	KC343121	KC344089	KC343605	KC343847	KC343363
*D. neotheicola*	Dne_MNE1; PHO2	**PP803052**	**PP786220**	**PP803736**	**PP803716**	**PP803696**
*D. nothofagi*	BRIP 54801 ^T^	JX862530	KF170922	–	JX862536	–
*D. phaseolorum*	CBS 113425	KC343174	KC344142	KC343658	KC343900	KC343416
	CBS 127465; GJS 83-379	KC343177	KC344145	KC343661	KC343903	KC343419
*D. rudis*	CBS 109292; AR3422; WJ 1443 ^T^	KC843331	KC843177	–	KC843090	KC843146
	CPC 28425	MG281137	MG281310	MG281485	MG281658	MG281834
	Dru_IT1; D1	**PP803041**	**PP786208**	**PP803725**	**PP803705**	**PP803684**
	Dru_IT2; D14	**PP803046**	**PP786214**	**PP803730**	**PP803710**	**PP803685**
	Dru_IT3; D24	**PP803050**	**PP786218**	**PP803734**	**PP803714**	**PP803686**
*D. toxica*	CBS 534.93; ATCC 96741 ^T^	KC343220	KC344188	KC343704	KC343946	KC343462
*D. vaccinii*	CBS 160.32; IFO 32646 ^T^	AF317578	KC344196	KC343712	GQ250326	KC343470
	CBS 118571; G.C.A.Dvacc	KC343223	KC344191	KC343718	KC343949	KC343465
*Diaporthella corylina*	CBS 121124; AR 4131	KC343004	KC343972	KC343488	KC343730	KC343246

Ex-type, ex-epitype, isotype, holotype, ex-neotype isolates are marked by an upper T. New sequences generated in this study are in bold.

Phylogenetic analyses were performed based on maximum parsimony (MP) using the Tree-Bisection and Reconnection (TBR) algorithm, where gaps were treated as missing data. The robustness of the topology was evaluated by 1000 bootstrap replications (Felsenstein, 1985). Measures for the MP, including tree length (TL), consistency index (CI), retention index (RI), and rescaled consistency index (RC), were also calculated.

New sequences and the multi-locus alignment were deposited in GenBank (**Table 2**) and TreeBASE (http://purl.org/phylo/treebase/phylows/study/TB2:S31396S), respectively.

### 2.3 Fungal growth and sporulation at different temperatures

The mycelial growth of the fungal isolates was evaluated on PDA-containing Petri plates (5.5-cm diameter). Briefly, the PDA plates were inoculated with a mycelial plug (approximately 1 mm in diameter) extracted from the border of a colony grown on PDA for 10 days in a growth chamber at 20°C with a photoperiod of 12 h. After inoculation, plates were sealed with Parafilm (Pechiney Plastic Packaging Inc., Chicago, Illinois) and incubated at constant temperatures of 5, 10, 15, 20, 25, 30, and 35°C. Six plates were prepared for each isolate-temperature combination, and the experiments were conducted twice. Two perpendicular diameters of the fungal colony were measured every two days until the colony reached the edge of the plate. The colony growth rate was then expressed as cm per day.

The colony surface of three plates for each isolate-temperature combination was then gently washed twice with 6 mL of double-distilled water per washing. Briefly, after pouring water in a plate, the colony surface was gently rubbed with the help of a steel spatula to disperse the cirri produced by pycnidia into suspension and remove any mycelium that may have covered the pycnidia. The obtained suspension was then collected in a 15-mL Falcon tube after filtering through a double-layer gauze to retain the mycelium removed from the colony. Then, the colony surface was again rinsed with water, and the second suspension was also filtered and collected as before. Alpha and beta conidia were counted using a hemocytometer (Bürker, HBG, Giessen, Germany), and their quantities were expressed as numbers per cm^2^ of colony. Alpha and beta conidia were identified based on their morphological characteristics (Gomes et al., 2013; Guarnaccia et al., 2018).

Finally, the plates were photographed individually with a digital camera (Nikon Coolpix 5700), and the number of pycnidia was counted using the Microsoft Paint software for Windows Operating System (Paint 3D ver. 6.1907.29027.0, Microsoft Corporation, Redmond, Washington, USA). We used the “Brush” command and the icon for a circular dot with different colors for mature (i.e., with strongly pigmented walls) and immature (i.e., with non- or weakly pigmented walls) pycnidia (Somana et al., 2010). Their quantity was then expressed as the number of pycnidia per cm^2^ of fungal colony. The colony area was calculated using the colony diameters.

### 2.4 Pathogenicity analysis

The pathogenicity of the isolates was analyzed using both mycelium plugs and conidia of a subset of isolates (**Table 1**) used in the ecology study.

For mycelial inoculation (Kaliterna et al., 2012), portions of green, healthy shoots of length approximately 30 cm were cut from 12-year-old, potted plants cv. Barbera grown in the field at the University campus (Piacenza, Italy), which showed typical PCLS symptoms following artificial inoculation with *D. ampelina* in preliminary tests (*not shown*). The shoots were disinfected with 70% ethanol for 10 s and a small portion (approximately 4 mm long and 2 mm deep) was removed from the shoot surface using a sterile cork borer. A small mycelial plug (4 mm in diameter) was excised from the margin of actively growing fungal colonies grown on PDA for 7 days at 25°C.

The mycelium plug was then laid on the shoot wound and immediately wrapped with Parafilm to avoid desiccation (Thompson et al., 2011; Du et al., 2021). Eight shoots were inoculated with each fungal isolate, and eight shoots were not inoculated with any isolate. The non-inoculated shoots were wounded, inoculated with a PDA plug and covered with Parafilm. All shoots (inoculated and non-inoculated) were individually placed in flasks containing 200 mL of sterilized tap water, with the lower shoot part immersed in water. After 10 days of incubation at 25°C, the shoots were disinfected, and both external and internal lengths of tissue discoloration were recorded both above and below the inoculation site. Koch’s postulates were fulfilled by re-isolation of the infecting fungus by transferring three small pieces of symptomatic tissue from the edge of each lesion to PDA plates. The cultured samples of the re-isolated fungus were identified via morphological comparison with the original isolate. All the experiments were conducted twice.

For inoculation with conidia (Erincik et al., 2003), 2-year-old potted cuttings of cv. Barbera were pruned to obtain a single shoot and grown in field conditions. When the shoots had six fully expanded leaves, the top four leaves and the corresponding three internodes were inoculated by spraying a conidial suspension of each fungal isolates until runoff. The conidial suspensions were obtained from 30-day-old fungal colonies grown on PDA as described before, adjusted to 1 × 10^6^ alpha conidia/mL. Five cuttings were inoculated with each fungal isolate, and five cuttings were not inoculated for control. The non-inoculated cuttings were sprayed with water only. The inoculated and control cuttings were enclosed in moistened plastic bags to maintain a saturated atmosphere and placed in a growth chamber at 20°C with a 12-h photoperiod for 24 h to promote infection. Then, the cuttings were moved to a greenhouse for 15 days. The disease severity on individual leaves and internodes was assessed by using a modified EPPO scale, in which the disease severity is categorized from 0 (healthy) to 5 (more than 75% of affected area) as described in **Supplementary materials (Supplementary Figure S1)**. Each experiment was set up per a randomized complete block design and conducted twice.

### 2.5 Data analysis

The data related to morphological traits were subjected to analysis of variance (ANOVA) with fungi (species and isolates), temperature, and their interaction as fixed factors. The data on pathogenicity traits of the different fungi were also subjected to ANOVA. Since the trial did not show any significant effect in a preliminary analysis, the data collected in the two replicate trials were considered replicates. The numbers of pycnidia and conidia were transformed using the natural logarithm function before ANOVA, while the percent disease severity was derived using the arcsin function. The averages of the main factor “fungus” were compared using the Tukey Honestly Significant Difference (HSD) Test with P = 0.05. The analyses were carried out using the SPSS software (IBM SPSS Statistics, version 29).

The interactions between morphological traits and temperature were analyzed via non-linear regression. Since we were more interested in the differences in the temperature responses of the different fungi rather than the differences in their mycelial growth or sporulation capability, the data were rescaled by dividing each value at any temperature by the value obtained at optimal temperature. The rescaled data were then regressed against temperature. Different bell-shaped non-linear regression equations were compared based on Akaike’s Information Criterion (AIC). The following Bethe equation (Analytis, 1977) provided the smallest AIC values and was therefore considered the most suitable (Burnham and Anderson, 2002):


(1) $$\text{Y}={\left({\text{aTeq}}^{\text{b}}\left(1-\text{Teq}\right)\right)}^{\text{c}}$$

Here, Y is the rescaled morphological trait (on a 0 to 1 scale); Teq is the equivalent temperature, calculated as (T − Tmin)/(Tmax − Tmin), where T is the temperature regime (°C), and Tmin and Tmax are minimal and maximal temperatures for mycelial growth, which were considered as equation parameters; and a, b, and c are the equation parameters defining the top, symmetry, and size of the unimodal curve, respectively. These equations were calculated for single *Diaporthe* spp. and not for single isolates within a species, whose variability is expressed by the standard error of equation parameter estimates and by whiskers in figures showing the curve fitting.

## 3 Results

### 3.1 Molecular identification of fungal isolates

**Figure 1:**
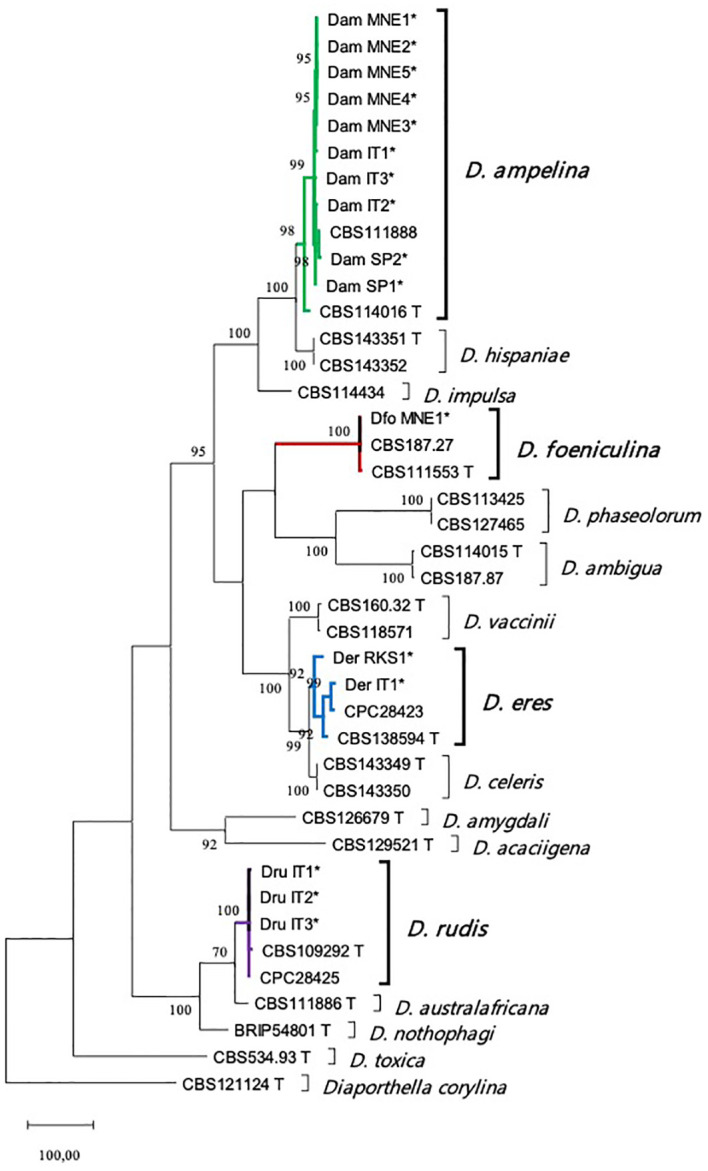
Maximum parsimony (MP) tree of the isolated *Diaporthe* species and their phylogenetically closely related species based on combined ITS, *tef1-α*, *tub*, *his*, and *cal* loci. Parsimony bootstrap support values for MP ≥ 70% are indicated above the nodes. The tree is rooted with *Diaporthella corylina* (CBS 121124). Ex-type and ex-epitype cultures are marked with a T. The isolates from this study are indicated by *. ITS, internal transcribed spacer; *tub*, beta-tubulin gene; *tef1-α*, translation elongation factor 1-alpha; *his*, histone H3; *cal*, calmodulin.

Five loci (the ITS region, partial *tub*, *tef1-α*, *cal*, and *his3*) were sequenced and deposited in GenBank (**Table 2**). A multi-locus analysis was performed with the *Diaporthe* spp. that were phylogenetically closely related to the isolates from the current study. The concatenated alignment for the five loci contained 2318 positions in the final dataset (519, 426, 364, 515, and 494 from ITS, *tef1-a*, *tub*, *his* and *cal*, respectively), with 1065 constant and 819 parsimony informative. The analysis included one outgroup (*Diaporthella corylina*, CBS121124) and 64 ingroup taxa (16 isolates obtained in the current study and 48 *Diaporthe* reference species). The MP analysis yielded the five most parsimonious trees (tree length = 3103, consistency index = 0.567, and retention index = 0.86). One of these trees is presented in **Figure 1**. The MP phylogeny showed that the sequences of the isolates from the present study fell into the clades corresponding to the known species *D. ampelina* (n = 10), *D. eres* (n = 2), *D. foeniculina* (n = 1), and *D. rudis* (n = 3).

### 3.2 Inter-isolate differences in growth and sporulation

**Figure 2:**
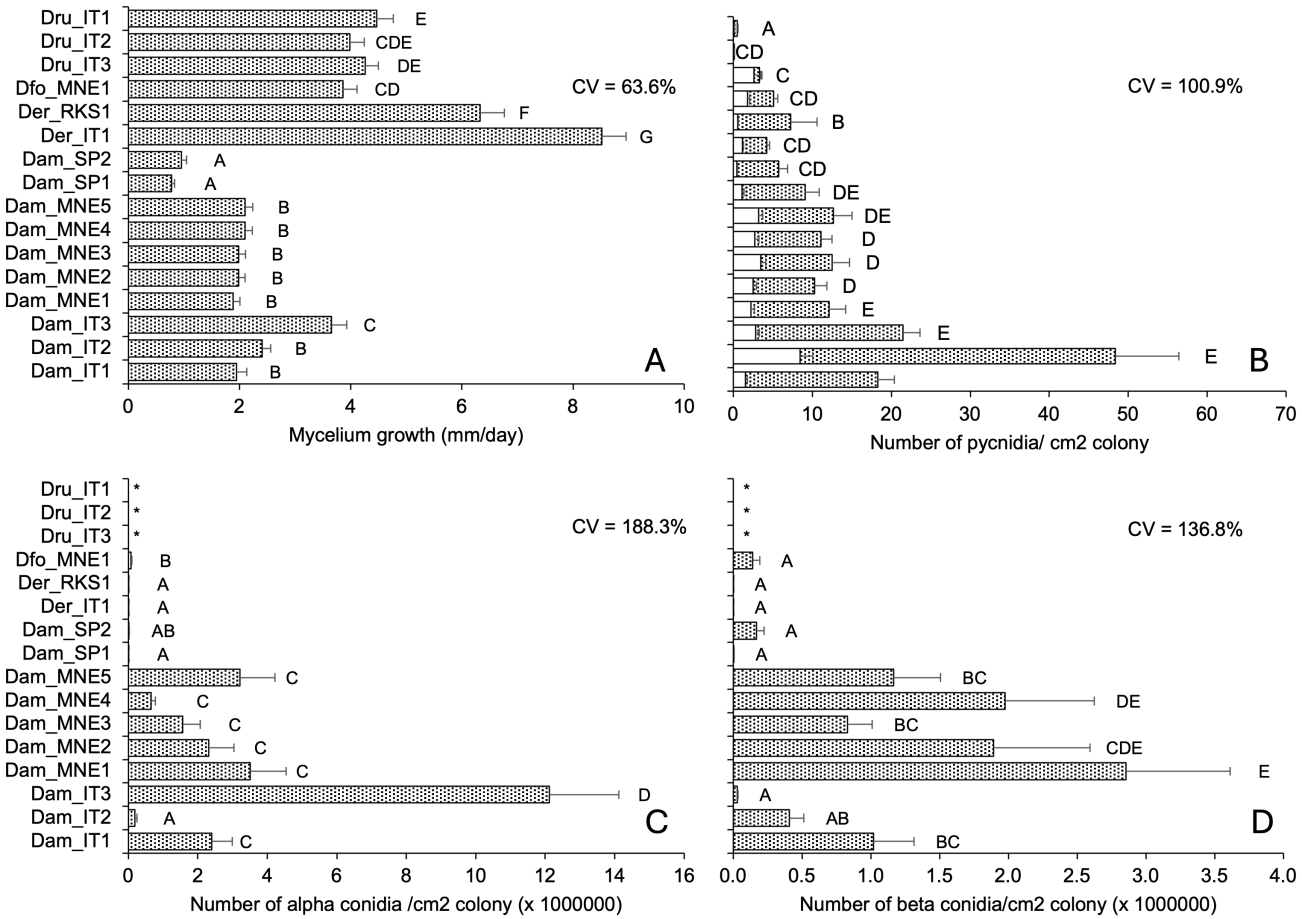
Morphological traits of 16 isolates of *Diaporthe ampelina*, *D. eres*, *D. foeniculina*, and *D. rudis* isolated from grapevine. Bars and error bars represent the average and standard error, respectively, for 84 parameters (seven, six, and two temperature regimes, replicates, and repeated experiments, respectively) related to mycelial growth **(A)** and 36 parameters (six, three, and two temperature regimes, replicates, and repeated experiments, respectively) for the production of pycnidia **(B)** and conidia **(C, D)**. Letters show significant differences based on Tukey’s test (P = 0.05), and CV indicates the coefficient of variation among averages. * indicates no conidia.

Isolate, temperature, and their interactions significantly impacted (P < 0.001) colony growth rate and production of pycnidia and alpha and beta conidia. We found differences between fungal species and isolates within a species, irrespective of the geographical origin of the isolate (**Figure 2**).

Isolates of *D. rudis*, *D. eres*, and *D. foeniculina* exhibited a faster mycelial growth than the *D. ampelina* isolates, with average growth rates of 5.24 for the former three species and 1.98 mm/day for the latter (**Figure 2A**). The *D. rudis* isolates produced very few pycnidia, with an average of 1.3 pycnidia/cm^2^ colony, and one of these isolates (*Dru*_IT2) only produced immature pycnidia (**Figure 2B**). *D. eres* and *D. foeniculina* produced a lower number of pycnidia than *D. ampelina*, with an average of 5.5 and 16.2 pycnidia/cm^2^ colony, respectively (**Figure 2B**). The proportions of mature pycnidia over total pycnidia for *D. ampelina*, *D. rudis*, *D. foeniculina*, and *D. eres* isolates were 72–93%, 22–73%, 65%, and 73–93%, respectively. Neither alpha nor beta conidia were produced by *D. rudis* isolates (**Figures 2C, D**) that produced very few, mainly immature pycnidia (**Figure 2B**). *D. foeniculina* isolates and the two Spanish isolates of *D. ampelina* also produced very few conidia (**Figures 2C, D**). Interestingly, isolate *Dam*_IT2 produced high levels of alpha and few beta conidia (**Figures 2C, D**). The inter-isolate variability, measured as coefficient of variation (CV), was the highest for conidia production, followed by pycnidia production and mycelial growth (**Figure 2**).

For all isolates, we observed a significant relationship between mycelial growth and pycnidia production, but no significant correlation was observed with the production of either alpha or beta conidia (**Table 3**). In addition, the proportion of mature pycnidia weakly correlated with the production of both alpha and beta conidia (r = 0.564 and 0.512, respectively). However, the production of both conidial types correlated well with each other (r = 0.958, **Table 3**).

**Table 3:** Pearson’s coefficients of correlation between the morphological traits of 16 fungal stains belonging to *Diaporthe ampelina*, *D. eres*, *D. foeniculina*, and *D. rudis* isolated from grapevine.

Morphological trait		(1)	(2)	(3)	(4)	(5)	(6)
Mycelial growth	(1)	1	0.816	0.507	0.487	0.444	0.398
			*<0.001*	*0.045*	*0.056*	*0.085*	*0.127*
Pycnidia production	immature (2)		1	0.815	0.798	0.595	0.541
				*<0.001*	*<0.001*	*0.015*	*0.03*
	mature (3)				0.960	0.564	0.512
				1	*<0.001*	*0.023*	*0.043*
	total (4)				1	0.606	0.553
						*0.013*	*0.026*
Conidia production	alpha (5)					1	0.958
							*<0.001*
	beta (6)						1

### 3.3 Effects of temperature on fungal growth and sporulation

The isolate-temperature interaction significantly impacted the fungal morphological traits. These interactions accounted for 27.1%, 18.6%, 24.8%, and 31.9% of the total experimental variance for mycelial growth, pycnidia production, alpha conidia production, and beta conidia production, respectively. Therefore, different fungi exhibited varying behaviors at different temperatures. Equation 1 provided a good fit of experimental data for all the fungi and their morphological traits, with R^2^ > 0.8 and low standard errors of parameter estimates (**Table 4**).

**Table 4:** Parameters of the Bethe equation (Equation 1 in the main text) fitting the temperature responses in terms of mycelial growth and production of pycnidia and alpha and beta conidia in four *Diaporthe* spp. isolated from grapevine.

Cardinal temperatures^a^				Equation parameters and statistics						
Fungus	Tmin	Topt	Tmax	a	es(a)^b^	b	es(b)	c	es(c)	R^2^
Mycelial growth										
*D. ampelina*	2.8	25.1	34.7	6.882	0.396	2.323	0.158	0.913	0.141	0.988
*D. rudis*	2.3	19.0	35.0	4.005	0.170	1.043	0.054	2.253	0.284	0.984
*D. eres*	3.2	24.2	37.0	5.562	0.902	1.632	0.270	1.183	0.399	0.960
*D. foeniculina*	4.1	26.3	35.0	8.005	0.843	2.565	0.305	1.010	0.446	0.960
Production of pycnidia										
*D. ampelina*	3.0	20.7	35.0	4.430	0.283	1.24	0.096	3.360	0.770	0.956
*D. rudis*	5.0	13.7	30.0	2.441	0.217	0.536	0.06	3.456	1.033	0.946
*D. eres*	4.0	17.3	35.0	2.752	0.530	0.755	0.161	1.236	0.463	0.883
*D. foeniculina*	5.0	16.2	37.0	2.71	0.200	0.542	0.066	4.474	1.388	0.915
Production of alpha conidia^c^										
*D. ampelina*	13.5	21.6	31.0	3.550	0.069	0.868	0.019	2.787	0.196	0.988
*D. eres*	10.5	16.9	30.0	2.325	0.051	0.488	0.023	1.374	0.098	0.999
*D. foeniculina*	11.0	16.9	30.0	2.722	0.042	0.543	0.014	26.45	1.121	0.999
Production of beta conidia^c^										
*D. ampelina*	11.0	27.5	35.0	5.119	0.293	2.186	0.147	1.090	0.181	0.994
*D. eres*	11.2	27.0	35.0	4.600	0.405	2.000	0.123	0.700	0.900	0.809
*D. foeniculina*	11.0	19.3	29.0	3.600	0.332	0.850	0.031	7.150	0.932	0.952

a Tmin, Topt, and Tmax are minimal, optimal, and maximal temperatures, respectively.

b es is the standard error of three estimated parameters.

c *D. rudis* did not produce alpha or beta conidia.

**Figure 3:**
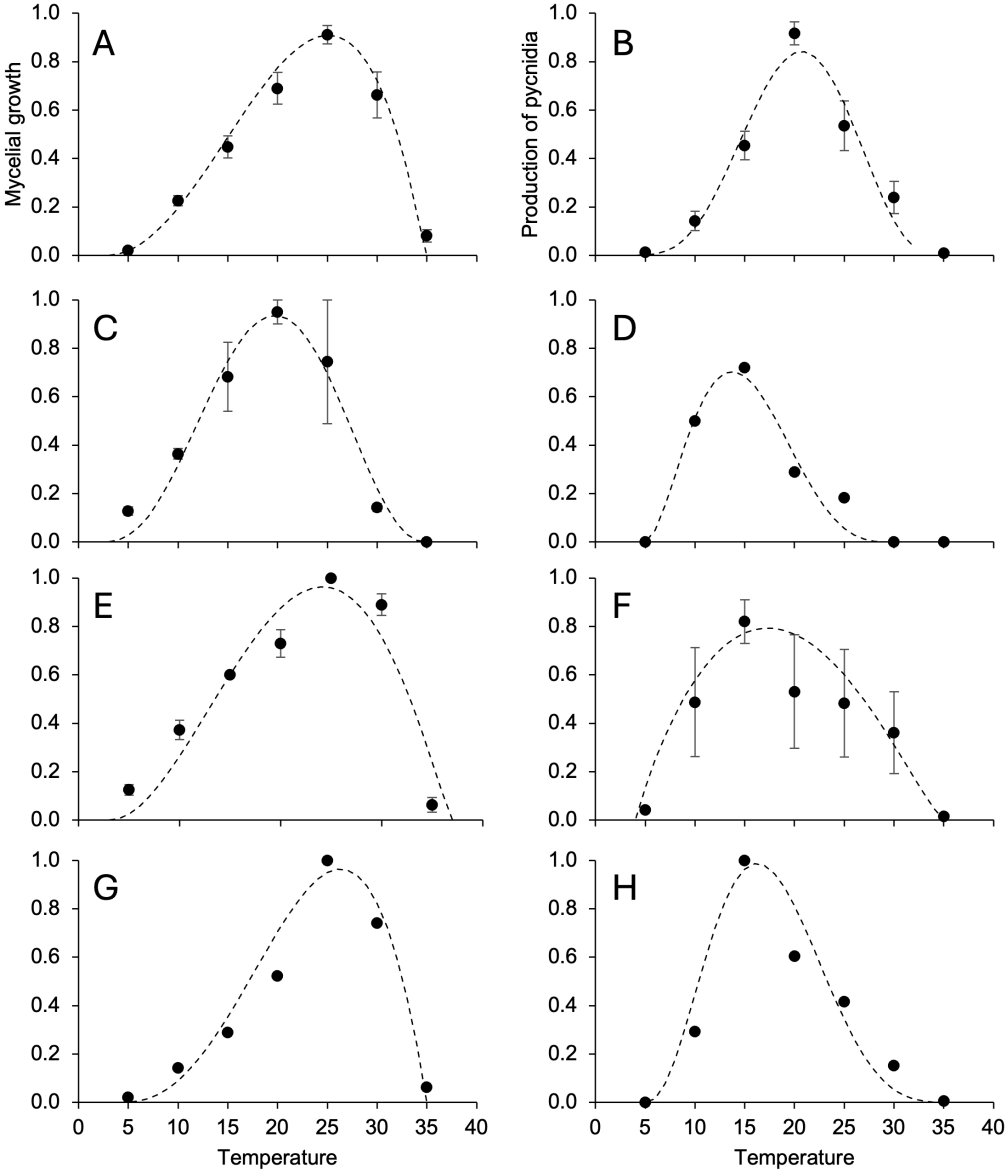
Temperature responses in terms of mycelial growth **(A, C, E, G)** and the production of pycnidia **(B, D, F, H)** in *Diaporthe ampelina* **(A, B)**, *D. rudis* **(C, D)**, *D. eres* **(E, F)**, and *D. foeniculina* **(G, H)** isolated from grapevine. Dots and error bars represent the average and standard error, respectively, for different isolates per species, as indicated in **Table 1**. The dotted lines show the Betes equation (Equation 1, see main text) fitting the data (see **Table 4** for equation parameters and statistics).

The mycelium of *D. rudis* (**Figure 3C**) and *D. eres* (**Figure 3E**) grew faster at 5°C than *D. ampelina* (**Figure 3A**) and *D. foeniculina* (**Figure 3G**), with optimal growth at lower temperatures (**Table 4**). No or minimal mycelial growth was observed at 35°C for all fungi. The temperature response was almost symmetrical around the optimal temperature (Topt) for *D. rudis* (Topt = 19°C) (**Figure 3C**) and negatively skewed for the other species, with Topt being closer to Tmax than Tmin (**Table 4**). Inter-isolate variability was high for some central temperature for *D. rudis* (**Figure 3C**).

The temperature range for pycnidia production was narrower than that for mycelial growth for all fungi, and Topt were lower by 4 to 10°C depending on the species (**Table 4**). No or very few pycnidia (<1/cm^2^ colony) were produced at 5°C. Some conidia (average = 4.5/cm^2^ colony) were produced at 30°C but not at 35°C. With respect to the mycelial growth, temperature response patterns varied across different *Diaporthe* spp. (**Figures 3B, D, F, H**), with Topt ranging between 13.7°C (for *D. rudis*) and 20.7°C (for *D. ampelina*) (**Table 4**). Inter-isolate differences, however, were high between *D. rudis* and *D. eres*, as demonstrated by the size of standard errors in **Figures 3D, F**.

**Figure 4:**
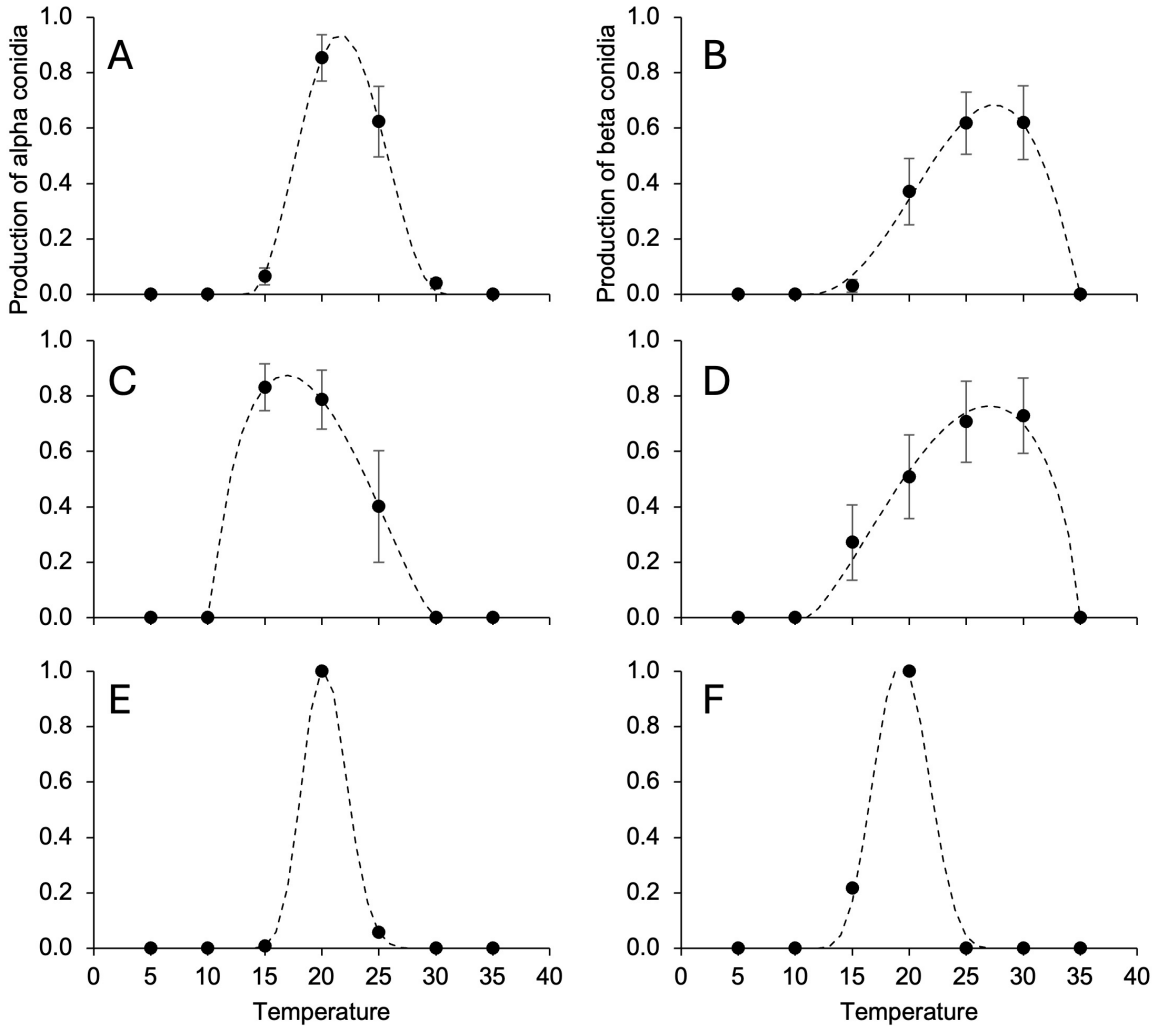
Temperature responses in terms of the production of alpha **(A, C, E)** and beta conidia **(B, D, F)** in *Diaporthe ampelina* **(A, B)**, *D. eres* **(C, D)**, *D. foeniculina* **(E, F)** isolated from grapevine. Dots and error bars represent the average and standard error, respectively, for different isolates per species, as indicated in **Table 1**. The dotted lines show the Betes equation (Equation 1, see main text) fitting the data (see **Table 4** for equation parameters and statistics).

The temperature range for alpha conidia production was narrower than that for pycnidia production, with minimal and maximal temperatures being >10°C and close to 30°C, respectively (**Table 4**). Topt for beta conidia production were higher than those for alpha conidia production (**Table 4**) and reflected different temperature responses for *D. ampelina* (**Figures 4A, B**) and *D. eres* (**Figures 4C, D**). The alpha and beta conidia production patterns were similar for *D. foeniculina* (**Figures 4E, F**).

### 3.4 Pathogenicity on shoots and leaves

**Figure 5:**
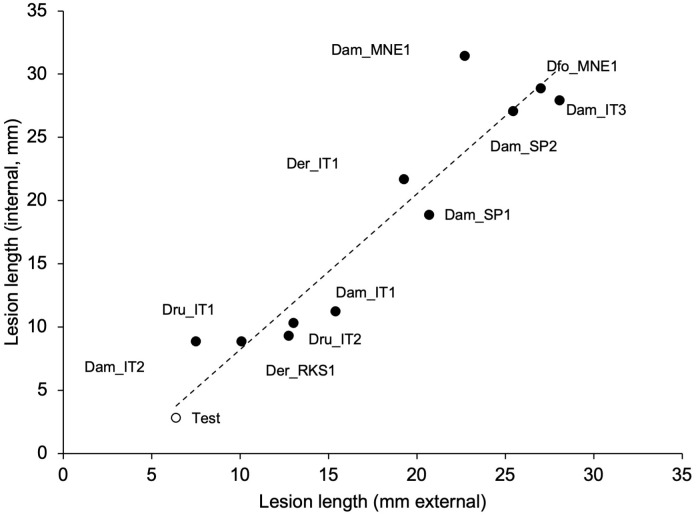
Relationship between the lengths of external and internal discolorations (lesions) caused by artificial inoculation of green grapevine shoot with mycelial plugs colonized by 11 isolates of *Diaporthe ampelina*, *D. eres*, *D. foeniculina*, and *D. rudis* (see **Table 1**) (full dots), or mock-inoculated (test, white dot). The dotted line shows the linear regression fitting the data: y = 1.23x − 4.07; R² = 0.896.

**Figure 6:**
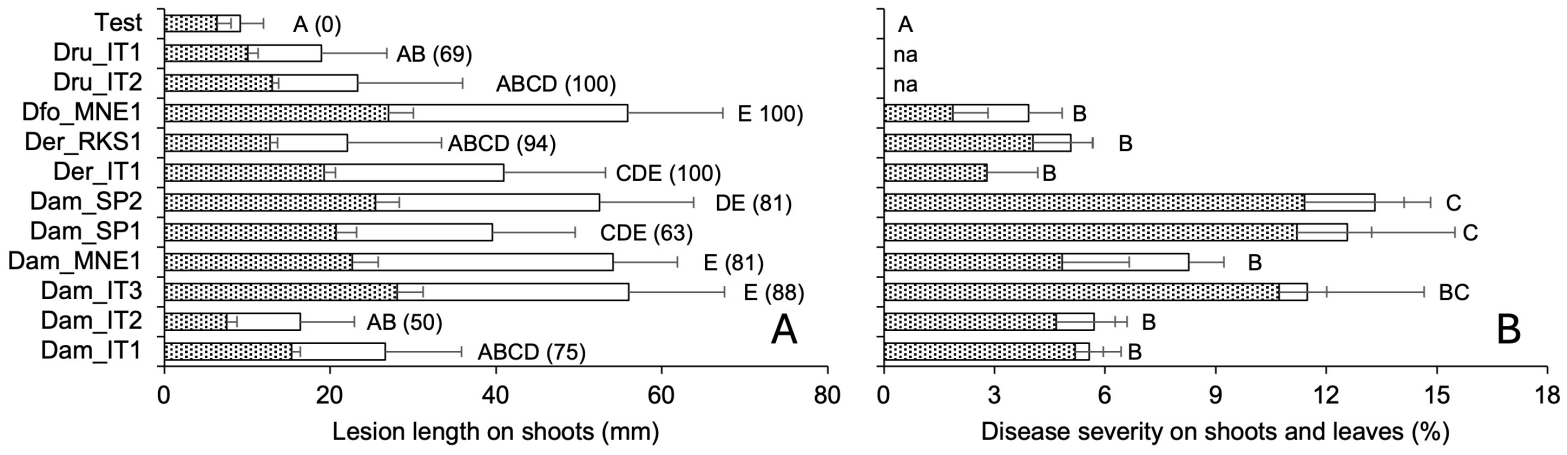
Pathogenicity traits of 11 isolate of *Diaporthe ampelina*, *D. eres*, *D. foeniculina*, and *D. rudis* isolated from grapevine. **(A)** Bars and errors bars represent the average and standard error, respectively, of lesion length on the external (white bars) and internal (dotted bars) tissue of green grapevine shoots that have been inoculated with mycelial plugs or mock-inoculated (six replicates and two repeated experiments). **(B)** Bars and error bars represent the average and standard error, respectively, of PCLS severity in green shoots (dotted bars) and leaves (white bars) inoculated with a conidial suspension or mock-inoculated. Letters show significant differences based on Tukey’s test (P = 0.05); in **(A)**, the numbers in parentheses represent the percentage of re-isolation from lesions produced after artificial inoculation.

The mycelial and conidial inoculations from different fungal species exhibited significantly different pathogenicity (P < 0.001). The mycelial inoculation of shoots resulted in both external and internal discoloration, with closely correlated lesion length (**Figure 5**). Some discoloration was also observed in 50% of the shoots inoculated with only agar; even though the intensity of discoloration was generally lower (**Figure 6A**). All the fungi induced discoloration in all the inoculated shoots, with high variability in lesion length and in differences between internal and external lesion length (**Figure 6A**). The percentage of re-isolation of the fungi samples from the lesions was overall high (**Figure 6A**). As a consequence, only some fungi were significantly different from the non-inoculated samples, including *D. foeniculina*, one Italian isolate of *D. eres* (*Der*_IT1), and four *D. ampelina* isolates (the two Spanish isolates, one isolate from Montenegro (*Dam*_MNE1), and one Italian isolate (*Dam*_IT3)) (**Figure 6A**).

**Figure 7:**
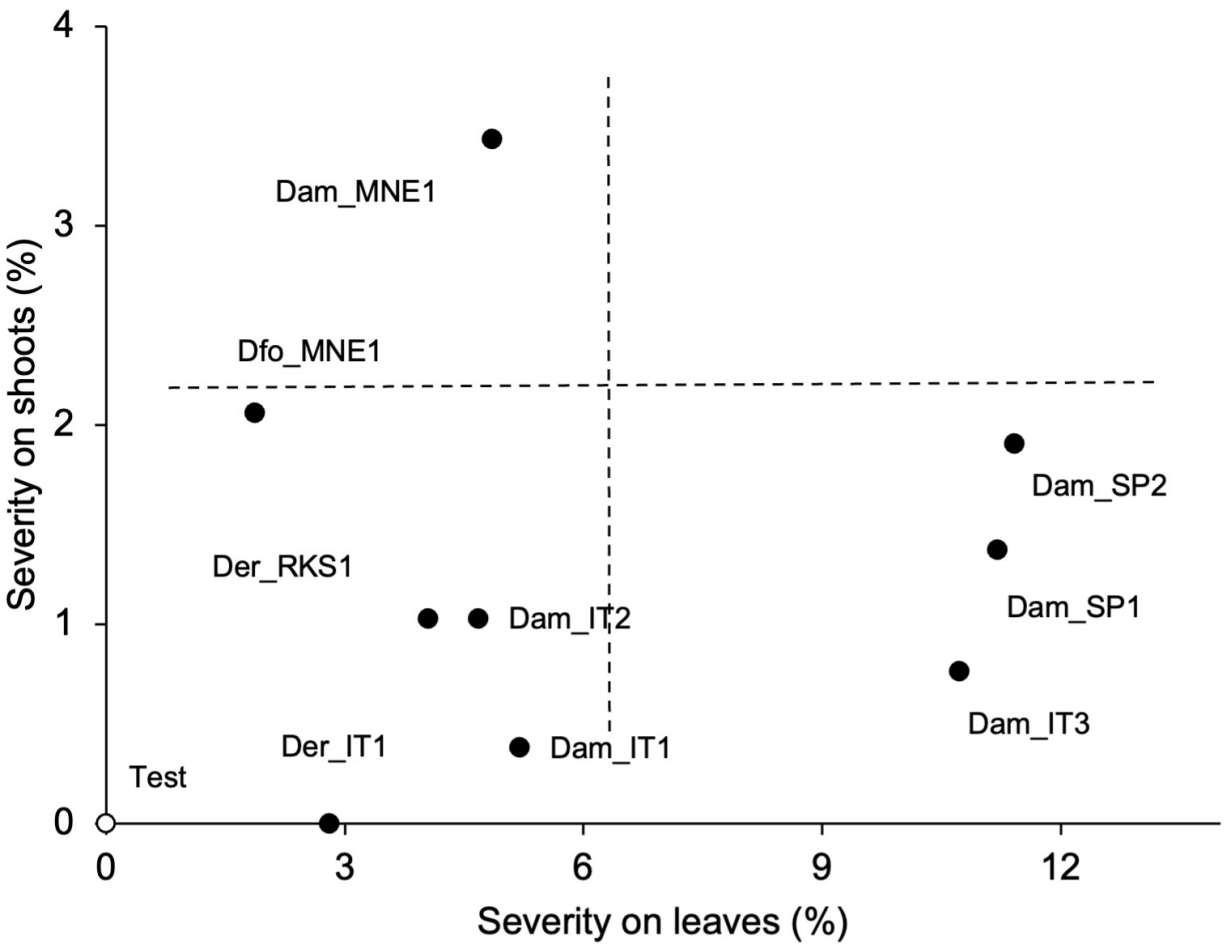
Relationship between the severity of PCLS symptoms on grapevine leaves and shoots artificially inoculated with conidia of nine isolates of *Diaporthe ampelina*, *D. eres*, *D. foeniculina*, and *D. rudis* (see **Table 1**; full dots) or mock-inoculated (test, white dot). Dotted lines split the area into four quadrants characterized by different combinations of disease severities on shoots and leaves.

Furthermore, we observed significant differences between the non-inoculated shoots/leaves and the shoots/leaves inoculated with fungal conidia (**Supplementary Figure S2**). The shoots/leaves inoculated with the conidia from the Spanish isolates of *D. ampelina* exhibited the highest overall disease severity, followed by the Italian isolate *Dam*_IT3 (**Figure 6B**). Furthermore, these isolates exhibited a higher pathogenicity on leaves than on shoots. In contrast, the two isolates from Montenegro (one each of *D. ampelina* and *D. foeniculina*) were more pathogenic on leaves than on shoots (**Figure 7**).

## 4 Discussion

In this study, we compared different *Diaporthe* isolates obtained from grapevine canes showing typical PCLS symptoms in Mediterranean, European countries with the ultimate goal to understand (i) whether other *Diaporthe* spp. are involved in the PCLS etiology, and (ii) whether these species have different responses to temperature, which may be considered in prediction models for PCLS (Gonzalez-Dominguez et al., 2021).

The multi-locus DNA sequence analyses revealed the presence of four species isolated from symptomatic tissues, namely *D. ampelina*, *D. eres*, *D. foeniculina*, and *D. rudis*. These species have already been found in the infected grapevine wood in previous studies as mentioned in the Introduction, though not from 1-year-old canes showing typical PCLS symptoms and bearing abundant pycnidia.

Even though this work was not designed as a survey to determine the frequency of the different *Diaporthe* spp. associate with PCLS symptoms in grapevine canes, *D. ampelina* was the most frequently isolated fungal species in the current study, so supporting its predominant role in PCLS. In addition, it was found to be the most aggressive in the pathogenicity analyses, which was in agreement with previous studies (Kaliterna et al., 2012; Úrbez-Torres et al., 2013; Baumgartner et al., 2013; Lawrence et al., 2015; Lesuthu et al., 2019). A high variability was observed among the *D. ampelina* isolates with respect to the severity of disease symptoms on both green shoots and leaves, indicating a high intra-specific variability, corroborating the findings from previous studies (Schilder et al., 2005; Kaliterna et al., 2012; Agkül and Awan, 2022).

In the present study, *D. foeniculina* isolated from the canes with typical PCLS symptoms was found to be pathogenic after artificial inoculation of both shoots and leaves, with a disease severity similar to those of some isolates of *D. ampelina*. *D. foeniculina* was initially isolated from fennel (*Foeniculum vulgare*) and was later found to be associated, as an opportunistic pathogen, with multiple host plants ranging from crops to temperate woody plants and fruit trees (Gomes et al., 2013; Udayanga et al., 2014; Sessa et al., 2017). In Croatia (Kaliterna et al., 2012), California (Úrbez-Torres et al., 2013; Lawrence et al., 2015), and South Africa (White et al., 2011; Lesuthu et al., 2019), *D. foeniculina* was isolated from grapevines affected by GTDs and considered a weak pathogen or an endophyte colonizing grapevine wood. It has also been found to cause shoot blight of persimmon (*Diospyros kaki*) (Golzar et al., 2012), cankers on shoots of kiwifruit (*Actinidia deliciosa*) (Thomidis et al., 2013), and branch dieback and shoot blight of English walnut (*Juglans regia*) (Lopez-Moral et al., 2022).

The discrepancies between the findings from our pathogenicity analyses and the results of previous studies might be attributed to the origin of the isolates (i.e., the plant tissue and disease symptom) and the artificial inoculation methods. For instance, Kaliterna et al. (2012) isolated *D. foeniculina* from diseased grapevine wood samples with GTD-related symptoms and inoculated the mycelium plugs on wounded green shoots and lignified canes. Lawrence et al. (2015) isolated the fungi samples from wood cankers and inoculated either mycelial fragments in suspension or alpha conidia on wounds made with powder drill on lignified canes. Úrbez-Torres et al. (2013) isolated *D. foeniculina* from perennial cankers on cordons or trunks from grapevines showing characteristic dieback symptoms and inoculated the mycelium plugs into the holes in mature wood cordon tissue wounded with a drill. In contrast, our isolate was obtained from 1-year-old grapevine canes with severe PCLS symptoms and abundant pycnidia. To the best of our knowledge, this was the first study to demonstrate the ability of *D. foeniculina* to cause PCLS in grapevine.

Furthermore, we also isolated *D. eres* from the 1-year-old grapevine canes. This species has previously been isolated from grapevine canes from vineyards in Italy, with bleached areas covered by black pycnidia, sometimes surrounded by dark margins and irregular dark blotches (Cinelli et al., 2016). Another study confirmed the pathogenicity of this species, along with its ability to produce metabolites with phytotoxic activity (Reveglia et al., 2021). It is highly polyphagous and has been described as pathogenic to many woody plant species (Anagnostakis, 2007; Thomidis and Michailides, 2009; Vrandečić et al., 2011). Earlier, it was reported as a moderately aggressive (compared to *D. ampelina*) pathogen on green shoots and lignified canes of grapevine (Kaliterna et al., 2012) and on woody stems of potted *V. labruscana* and *V. vinifera* (Baumgartner et al., 2013). Later, it was isolated from the vineyards in California (Úrbez-Torres et al., 2013), Europe (Guarnaccia et al., 2018), South Africa (Lesuthu et al., 2019), and China, where it was identified as the dominant *Diaporthe* sp. infecting grapevine (Dissanayake et al., 2015; Manawasinghe et al., 2019). *Diaporthe eres* was also isolated from late-season bunch rots of wine grapes in the Mid-Atlantic region of the US, together with *D. ampelina* and *D. guangxiensis*, and all these species were found to be aggressive when inoculated on detached berries of both table and wine grapes (Cosseboom and Hu, 2023).

Interestingly, *D. eres* isolates were also recovered from severely diseased bunches of withered grapes for Amarone wine production in northern Italy, and pathogenicity tests revealed that this species infects berries and causes fruit rot (Lorenzini and Zapparoli, 2018). In our pathogenicity analyses, the *D. eres* isolates showed similar pathogenicity but lower than the pathogenicity of *D. foeniculina* and some isolates of *D. ampelina*. Nevertheless, in agreement with the results of Kaliterna et al. (2012), *D. eres* should be considered one of the causal agents of PCLS on grapevine.

*D. rudis* has previously been isolated from different host plants across Canada, Europe, New Zealand, South America, and South Africa, including grapevine (Guarnaccia et al., 2018). In Europe, the species has been isolated from the vineyards of the Czech Republic, France, Italy, Spain, and UK, confirming its association with grapevine (Guarnaccia et al., 2018). This species has been found to contribute, together with other pathogenic fungi, to the decay of grapes during withering (Lorenzini and Zapparoli, 2019). In the current study, *D. rudis* mycelia were non-pathogenic on green shoots, while conidial inoculation was not possible since we found no conidia production for this species, contrary to the findings of Udayanga et al. (2014). Therefore, the role of *D. rudis* in PCLS needs further exploration.

Overall, based on our results and the findings from previous studies, PCLS etiology needs to be reconsidered. Even though *D. ampelina* is undoubtedly the most important causal agent of this disease, other *Diaporthe* spp., namely *D. foeniculina* and *D. eres*, should be considered as part of a complex of species causing PCLS when inoculated on green shoots and leaves of grapevines. This involves that the range of environmental conditions favorable for PCLS development should be widened. Indeed, optimal temperature ranges varied among the three species, with *D. rudis* showing the lower optimal temperatures for both mycelial growth and production of pycnidia, and *D. foeniculina* and *D. eres* producing pycnidia and alpha conidia at lower temperatures than *D. ampelina*. In agreement with our results, in spore-trapping studies during the dormant season in California, *D. ampelina* was rarely found, differently from conidia of *D. chamaeropis*, *D. eres*, and *D. foeniculina*, which were very common, indicating that the latter species may have cooler temperature requirements for spore production than *D. ampelina* (Fujiyoshi et al., 2021). Unpublished spore trapping studies in Michigan and New York found differences in alpha and beta conidia abundance along the season, with beta conidia being more frequent later in the growing season (Wilcox et al., 2015). This is consistent with our results, in which the optimal temperatures for the production of beta conidia by *D. ampelina* and *D. eres*. Future studies are need to ascertain whether these differences are also valid for conidia-mediated infection. If so, the model proposed by Gonzalez-Dominguez et al. (2021), which is currently parameterized for *D. ampelina*, would need to be optimized to include these additional *Diaporthe* spp.

## Data Availability

The datasets presented in this study can be found in online repositories. The names of the repository/repositories and accession number(s) can be found in the article/**Supplementary Material**.

## References

[B1] Akgül D. S. Awan Q. N. (2022). Characterization of *Diaporthe ampelina* isolates and their Sensitivity to Hot-Water Treatments and Fungicides in *in vitro* . Kahramanmaraş. Sütçü. İmam. Üniversitesi. Tarım. ve. Doğa. Dergisi. 25, 1378–1389. doi: 10.18016/ksutarimdoga.vi.1020144

[B2] Anagnostakis S. L. (2007). *Diaporthe eres* (*Phomopsis oblonga*) as a pathogen of butternut (*Juglans cinerea*) in Connecticut. Plant Dis. 91, 1198. doi: 10.1094/PDIS-91-9-1198C 30780664

[B3] Analytis S. (1977). On the relation between biological development and temperature of some plant pathogenic fungi. J. Phytopathol. 90, 64–76. doi: 10.1111/j.1439-0434.1977.tb02886.x

[B4] Baumgartner K. Fujiyoshi P. T. Travadon R. Castlebury L. A. Wilcox W. F. Rolshausen P. E. (2013). Characterization of species of *Diaporthe* from wood cankers of grape in eastern North American vineyards. Plant Dis. 97, 912–920. doi: 10.1094/PDIS-04-12-0357-RE 30722541

[B5] Berrysmith F. (1962). ‘Dead arm’ disease of grapevines. New Z. J. Agric. 105, 309–313.

[B6] Burnham K. P. Anderson D. R. (2002). Model selection and multimodel interface (New York: Springer).

[B7] Carbone M. J. Gelabert M. Moreira V. Mondino P. Alaniz S. (2022). Grapevine nursery propagation material as source of fungal trunk disease pathogens in Uruguay. Front. Fungal Biol. 3. doi: 10.3389/ffunb.2022.958466 PMC10512308 37746215

[B8] Chamberlain G. C. Willison R. S. Townshend J. L. de Ronde J. H. (1964). Two fungi associated with the dead-arm disease of grape. Can. J. Bot. 42, 351–355. doi: 10.1139/b64-034

[B9] Cinelli T. Mondello V. Marchi G. Burruano S. Alves A. Mugnai L. (2016). First report of *Diaporthe eres* associated with cane blight of grapevine (*Vitis vinifera*) in Italy. Plant Dis. 100, 532–532. doi: 10.1094/PDIS-08-15-0872-PDN

[B10] Coleman L. C. (1928). The dead-arm disease of grapes in Ontario. A preliminary study. Sci. Agric. 8, 281–315.

[B11] Cosseboom S. D. Hu M. (2023). Identification and pathogenicity of cladosporium, fusarium, and diaporthe spp. Associated with late-season bunch rots of grape. Plant Dis. 107, 2929–2934. doi: 10.1094/PDIS-01-23-0146-SC 37005504

[B12] Dissanayake A. J. Liu M. Zhang W. Chen Z. Udayanga D. Chukeatirote E.. (2015). Morphological and molecular characterisation of *Diaporthe* species associated with grapevine trunk disease in China. Fungal Biol. 119, 283–294. doi: 10.1016/j.funbio.2014.11.003 25937058

[B13] Du Y. Wang X. Guo Y. Xiao F. Peng Y. Hong N.. (2021). Biological and molecular characterization of seven *Diaporthe* species associated with kiwifruit shoot blight and leaf spot in China. Phytopathol. Mediterr. 60, 177–198. doi: 10.36253/phyto-12013

[B14] Erincik O. Madden L. V. Ferree D. C. Ellis M. A. (2002). Infection of grape berry and rachis tissues by *Phomopsis viticola* . Plant Health Prog. 3. doi: 10.1094/PHP-2002-0702-01-RS 30823128

[B15] Erincik O. Madden L. V. Ferree D. C. Ellis M. A. (2003). Temperature and wetness-duration requirements for grape leaf and cane infection by *Phomopsis viticola* . Plant Dis. 87, 832–840. doi: 10.1094/PDIS.2003.87.7.832 30812895

[B16] Felsenstein J. (1985). Confidence limits on phylogenies: An approach using the bootstrap. Evolution;. Int. J. Organic. Evol. 39, 783–791. doi: 10.1111/j.1558-5646.1985.tb00420.x 28561359

[B17] Fujiyoshi P. T. Lawrence D. P. Travadon R. Cooper M. Verdegaal P. Schwebs S.. (2021). Detection of spores of causal fungi of dieback-type trunk diseases in young, asymptomatic vineyards and mature, symptomatic vineyards. Crop Prot. 150, 105978.

[B18] Golzar H. Tan Y. P. Shivas R. G. Wang C. (2012). First report of shoot blight of persimmon caused by *Diaporthe neotheicola* in Australia. Australas. Plant Dis. Notes 7, 115–117. doi: 10.1007/s13314-012-0061-y

[B19] Gomes R. R. R. Glienke C. Videira S. I. R. I. R. Lombard L. Groenewald J. Z. Z. Crous P. W. W. (2013). Diaporthe: A genus of endophytic, saprobic and plant pathogenic fungi. Persoonia 31, 1–41. doi: 10.3767/003158513X666844 24761033 PMC3904044

[B20] González-Domínguez E. Caffi T. Languasco L. Latinovic N. Latinovic J. Rossi V. (2021). Dynamics of *Diaporthe ampelina* conidia released from grape canes that overwintered in the vineyard. Plant Dis. 105, 3092–3100. doi: 10.1094/PDIS-12-20-2639-RE 33755509

[B21] Guarnaccia V. Groenewald J. Z. Woodhall J. Armengol J. Cinelli T. Eichmeier A.. (2018). Diaporthe diversity and pathogenicity revealed from a broad survey of grapevine diseases in Europe. Persoonia 40, 135–153. doi: 10.3767/persoonia.2018.40.06 30504999 PMC6146647

[B22] Kajitani Y. Kanematsu S. (2000). *Diaporthe kyushuensis* sp. *nov.*, the teleomorph of the causal fungus of grapevine swelling arm in Japan, and its anamorph *Phomopsis vitimegaspora* . Mycoscience 41, 111–114. doi: 10.1007/BF02464318

[B23] Kaliterna J. Miličević T. Cvjetković B. (2012). Grapevine trunk diseases associated with fungi from the Diaporthaceae family in Croatian vineyards. Arhiv. za. Higijenu. Rada. i Toksikologiju. 63, 471–479. doi: 10.2478/10004-1254-63-2012-2226 23334042

[B24] Kuo K. Leu L. S. (1998). *Phomopsis vitimegaspora*: A new pathogenic Phomopsis from vines. Mycotaxon 66, 497–499.

[B25] Lawrence D. P. Travadon R. Baumgartner K. (2015). Diversity of *Diaporthe* species associated with wood cankers of fruit and nut crops in northern California. Mycologia 107, 926–940. doi: 10.3852/14-353 26240309

[B26] León M. Berbegal M. Rodríguez-Reina J. M. Elena G. Abad-Campos P. Ramón-Albalat A.. (2020). Identification and characterization of *Diaporth*e spp. associated with twig cankers and shoot blight of almonds in Spain. Agronomy 10, 1062. doi: 10.3390/agronomy10081062

[B27] Lesuthu P. Mostert L. Spies C. F. J. Moyo P. Regnier T. Halleen F. (2019). *Diaporthe nebulae* sp. *nov.* and first report of *D. cynaroidis*, *D. novem*, and *D. serafiniae* on grapevines in South Africa. Plant Dis. 103, 808–817. doi: 10.1094/PDIS-03-18-0433-RE 30920350

[B28] López-Moral A. Lovera M. Antón-Domínguez B. I. Gámiz A. M. Michailides T. J. Arquero O.. (2022). Effects of cultivar susceptibility, branch age, and temperature on infection by Botryosphaeriaceae and Diaporthe fungi on English walnut (Juglans regia). Plant Dis. 106, 2920–2926. doi: 10.1094/PDIS-09-21-2042-RE 35380463

[B29] Lorenzini M. Zapparoli G. (2018). Identification of *Pestalotiopsis bicilita*, *Diplodia seriata* and *Diaporthe eres* causing fruit rot in withered grapes in Italy. Eur. J. Plant Pathol. 151, 1089–1093. doi: 10.1007/s10658-017-1416-1

[B30] Lorenzini M. Zapparoli G. (2019). *Diaporthe rudis* associated with berry rot of postharvest grapes in Italy. Plant Dis. 103, 1030–1103. doi: 10.1094/PDIS-10-18-1757-PDN

[B31] Manawasinghe I. S. Dissanayake A. J. Li X. Liu M. Wanasinghe D. N. Xu J.. (2019). High genetic diversity and species complexity of Diaporthe associated with grapevine dieback in China. Front. Microbiol. 10. doi: 10.3389/fmicb.2019.01936 PMC6732904 31543868

[B32] Merrin S. J. Nair N. G. Tarran J. (1995). Variation in Phomopsis recorded on grapevine in Australia and its taxonomic and biological implications. Australas. Plant Pathol. 24, 44–56. doi: 10.1071/APP9950044

[B33] Mostert L. Crous P. Petrini O. (2001). Endophytic fungi associated with shoots and leaves of *Vitis vinifera*, with specific reference to the *Phomopsis viticola* complex. Sydowia 52, 46–58.

[B34] Pearson R. C. Goheen A. C. (1994). “Phomopsis cane and leaf spot,” in Compendium of grape diseases. Eds. Hewitt W. B. Pearson R. C. (APS Publishing), 17–18.

[B35] Pine T. S. (1958). Etiology of the dead-arm. Phytopathology 48, 192–197.

[B36] Pine T. S. (1959). Development of the grape dead-arm disease. Phytopatho-. logy. 49, 738–743.

[B37] Pscheidt J. W. Pearson R. C. (1989). Time of infection and control of Phomopsis fruit rot of grape. Plant Dis. 73, 829–833. doi: 10.1094/PD-73-0829

[B38] Rawnsley B. Wicks T. J. Scott E. S. Stummer B. E. (2004). *Diaporthe perjuncta* does not cause Phomopsis cane and leaf spot disease of grapevines in Australia. Plant Dis. 88, 1005–1010. doi: 10.1094/PDIS.2004.88.9.1005 30812213

[B39] Reddick D. (1909). Necrosis of the grapevine. Cornell. Univ. Agric. Exp. Station Bull. 263, 323–343.

[B40] Reddick D. (1914). *Dead arm disease of grapes.* New York State Agriculture Experimental Station, Geneva, NY. Bull. 389, 463–490.

[B41] Reveglia P. Pacetti A. Masi M. Cimmino A. Carella G. Marchi G.. (2021). Phytotoxic metabolites produced by *Diaporthe eres* involved in cane blight of grapevine in Italy. Nat. Prod. Res. 35, 2872–2880. doi: 10.1080/14786419.2019.1679133 31674838

[B42] Schilder A. M. C. Erincik O. Castlebury L. Rossman A. Ellis M. A. (2005). Characterization of *Phomopsis* spp. infecting grapevines in the Great Lakes region of North America. Plant Dis. 89, 755–762. doi: 10.1094/PD-89-0755 30791247

[B43] Sessa L. Abreo E. Betucci L. Lupo S. (2017). Diversity and virulence of *Diaporthe* species associated with wood disease symptoms in deciduous fruit trees in Uruguay. Phytopathol. Mediterr. 56, 431–444.

[B44] Somana W. Burgett M. Warrit N. Sukumalanand P. (2010). A simplified technique using Microsoft Paint for counting cell numbers in honeybee and stingless bee colonies. Sci. Bee Culture. 2, 7–8.

[B45] Tamura K. Stecher G. Kumar S. (2021). MEGA11: Molecular evolutionary genetics analysis version 11. Mol. Biol. Evol. 38, 3022–3027. doi: 10.1093/molbev/msab120 33892491 PMC8233496

[B46] Thomidis T. Exadaktylou E. Chen S. (2013). *Diaporthe neotheicola*, a new threat for kiwi fruit in Greece. Crop Prot. 47, 35–40. doi: 10.1016/j.cropro.2012.12.024

[B47] Thomidis T. Michailides T. J. (2009). Studies on *Diaporthe eres* as a new pathogen of peach trees in Greece. Plant Dis. 93, 1293–1297. doi: 10.1094/PDIS-93-12-1293 30759511

[B48] Thompson J. D. Higgins D. G. Gibson T. J. (1994). Clustal W: Improving the sensitivity of progressive multiple sequence alignment through sequence weighting, position-specific gap penalties and weight matrix choice. Nucleic Acids Res. 22, 4673–4680. doi: 10.1093/nar/22.22.4673 7984417 PMC308517

[B49] Thompson S. M. Tan Y. P. Young A. J. Neate S. M. Aitken E. A. B. Shivas R. G. (2011). Stem cankers on sunflower (*Helianthus annuus*) in Australia reveal a complex of pathogenic *Diaporthe* (*Phomopsis*) species. Persoonia 27, 80–89. doi: 10.3767/003158511X617110 22403478 PMC3251322

[B50] Udayanga D. Castlebury L. A. Rossman A. Y. Hyde K. D. (2014). Species limits in *Diaporthe:* Molecular re-assessment of *D. citri*, *D. cytosporella*, *D. foeniculina* and *D. rudis* . Persoonia 32, 83–101. doi: 10.3767/003158514X679984 25264384 PMC4150081

[B51] Úrbez-Torres J. R. Adams P. Kamas J. Gubler W. D. (2009). Identification, incidence, and pathogenicity of fungal species associated with grapevine dieback in Texas. Am. J. Enol. Viticult. 60, 497–507. doi: 10.5344/ajev.2009.60.4.497

[B52] Úrbez-Torres J. R. Peduto F. Smith R. J. Gubler W. D. (2013). Phomopsis dieback: A grapevine trunk disease caused by *Phomopsis viticola* in California. Plant Dis. 97, 1571–1579. doi: 10.1094/PDIS-11-12-1072-RE 30716818

[B53] Úrbez-Torres J. R. Peduto F. Striegler R. K. Urrea-Romero K. E. Rupe J. C. Cartwright R. D.. (2012). Characterization of fungal pathogens associated with grapevine trunk diseases in Arkansas and Missouri. Fungal Diversity 52, 169–189. doi: 10.1007/s13225-011-0110-4

[B54] Vrandečić K. Jurković D. Ćosić J. Postić J. Riccioni L. (2011). First report of cane blight on blackberry caused by *Diaporthe eres* in Croatia. Plant Dis. 95, 612. doi: 10.1094/PDIS-11-10-0860 30731950

[B55] White C. Halleen F. Mostert L. (2011). Symptoms and fungi associated with esca in South Africa. Phytopathol. Mediterr. 50, S236–S246.

[B56] Wilcox W. F. Ellis M. A. Rawnsley B. Rossman A. Pscheidt J. (2015). “Phomopsis cane and leaf spot of grape,” in Compendium of grape disease, disorders, and pets, 2nd Ed. Wilcox W. F. Gubler W. D. Uyemoto J. K. (St. Paul, MN: American Phytopathological Society), 68–72.

