# Supplementary material for: Diaporthe foeniculina and D. eres, in addition to D. ampelina, may cause Phomopsis cane and leaf spot disease in grapevine
Source: Front Plant Sci. 2024 Sep 2;15:1446663. doi: 10.3389/fpls.2024.1446663 (PMC11402675; doi:10.3389/fpls.2024.1446663)
Supplement: Supplementary file 1 [file DataSheet1.docx]

**Supplementary materials**

**Figures**

Figure S1. Modified EPPO scale (EPPO, 2001) used for disease severity assessment on internodes (A) and individual leaves (B). The numerical rank corresponds to the following: 0= healthy with no lesions, 1= lesions covering 0-10% of the leaf or shoot surface, 2= lesions covering 10-30% of the surface, 3= lesions covering 30-50% of the surface, 4= lesions covering 50-75% of the surface, 5= lesions covering more than 75% of the surface. In categories 3 to 5, leaf blades can look distorted.

Figure S2. Example of typical PCLS symptoms following artificial inoculation on leaves (A), and grapevine shoots (B, C). Leaves were inoculated by spraying a conidial suspension and disease severity on individual leaves was assessed 15 days after. Shoots were inoculated with a mycelial plug (4 mm in diameter) laid on a shoot wound and wrapped with Parafilm. The non-inoculated shoots (D) were wounded, inoculated with a PDA plug and covered with Parafilm. After 10 days of incubation at 25 °C, both external (B) and internal (C) lengths of tissue discoloration on shoots were recorded both above and below the inoculation site (as indicated by the red arrows).
