# Supplementary figures and images for: Diaporthe foeniculina and D. eres, in addition to D. ampelina, may cause Phomopsis cane and leaf spot disease in grapevine
Source: Front Plant Sci. 2024 Sep 2;15:1446663. doi: 10.3389/fpls.2024.1446663 (PMC11402675; doi:10.3389/fpls.2024.1446663)

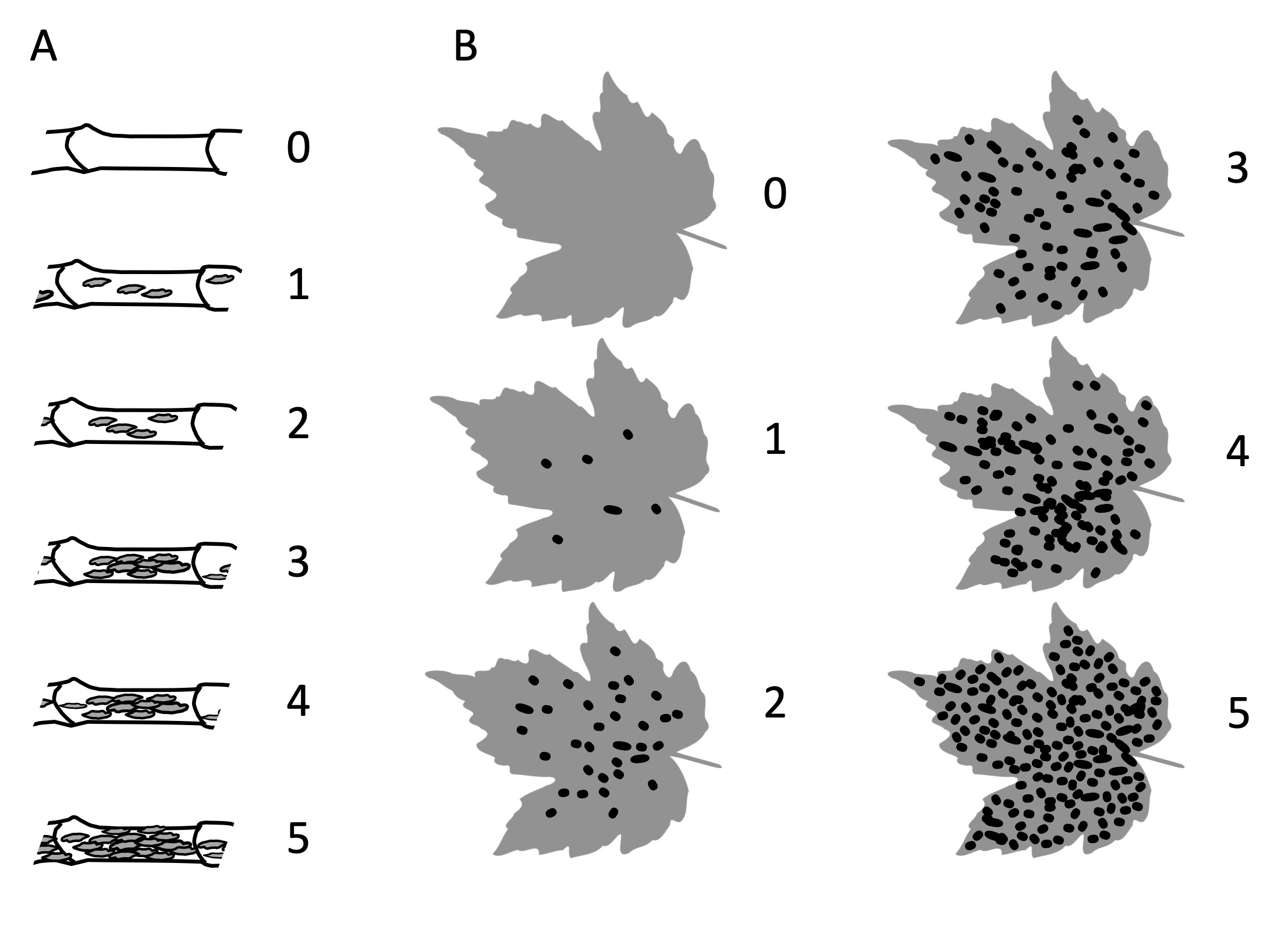

Supplement: Supplementary file 2 [file Image1.jpg]

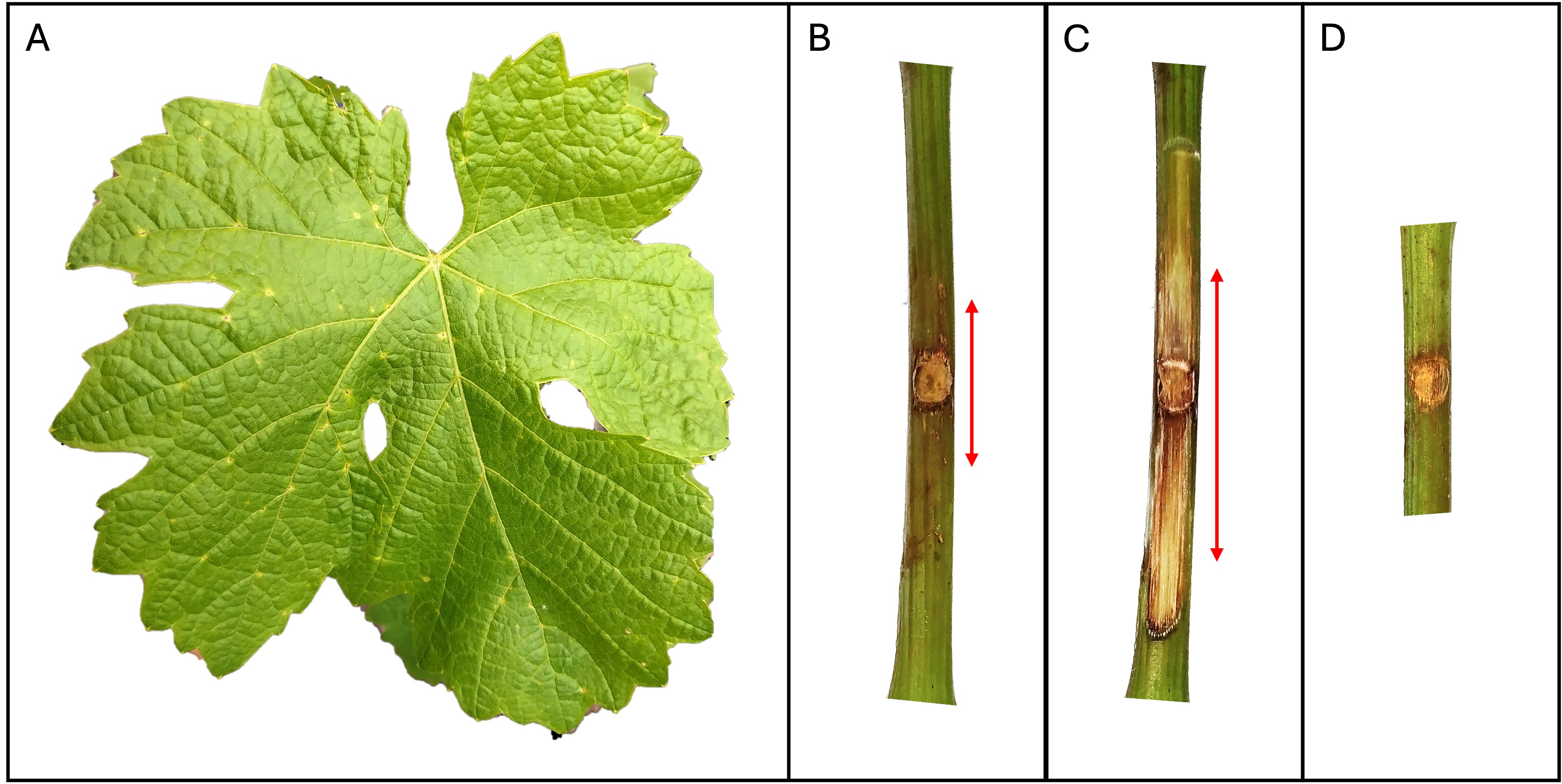

Supplement: Supplementary file 3 [file Image2.jpg]
