# Supplementary material for: Diaporthe foeniculina and D. eres, in addition to D. ampelina, may cause Phomopsis cane and leaf spot disease in grapevine
Source: Front Plant Sci. 2024 Sep 2;15:1446663. doi: 10.3389/fpls.2024.1446663 (PMC11402675; doi:10.3389/fpls.2024.1446663)
Supplement: Supplementary file 4 [file Table1.docx]

**Supplementary materials**

Table S1. Primers used for amplification and sequencing

| Locus | primer | Sequence | Reference |
| --- | --- | --- | --- |
| ITS | ITS4 | TCCTCCGCTTATTGATATGC | White et al. 1990 |
|  | ITS5 | GGAAGTAAAAGTCGTAACAAGG | White et al. 1990 |
| *tub2* | BT2a | GGTAACCAAATCGGTGCTGCTTTC | Glass and Donaldson, 1995 |
|  | BT2b | ACCCTCAGTGTAGTGACCCTTGGC | Glass and Donaldson, 1995 |
| *tef1-α* | EF1-688F | CGGTCACTTGATCTACAAGTGC | Alves et al. 2008 |
|  | EF1-1251R | CCTCGAACTCACCAGTACCG | Alves et al. 2008 |
| *his* | CYLH3F | AGG TCC ACT GGT GGC AAG | Crous et al. 2004 |
|  | H3-1b | GCGGGCGAGCTGGATGTCCTT | Glass and Donaldson, 1995 |
| *cal* | CAL-228F | GAGTTCAAGGAGGCCTTCTCCC | Carbone and Kohn, 1999 |
|  | CAL-737R | CATCTTTCTGGCCATCATGG | Carbone and Kohn, 1999 |
|  | CAL-563F | GACAAATCA CCACCAARGAGC | Udayanga et al. 2014 |
|  | CL2C | CTTCTGCATCATGAGCTGGAC | Weir et al. 2012 |

**References**

Alves, A., Crous, P. W., Correia, A., and Phillips, A. J. L. (2008). Morphological and molecular data reveal cryptic speciation in *Lasiodiplodia theobromae*. *Fungal Diversity*, *28*, 1–13.

Carbone, I., and Kohn, L. M. (1999). A method for designing primer sets for speciation studies in filamentous ascomycetes. *Mycologia*, *91*(3), 553–556. <https://doi.org/10.1080/00275514.1999.12061051>

Crous, P. W., Groenewald, J. Z., Risède, J. M., Simoneau, P., and Hywel-Jones, N. L. (2004). *Calonectria* species and their *Cylindrocladium* anamorphs: Species with sphaeropedunculate vesicles. *Studies in Mycology*, *50*, 415–430.

EPPO (2001). European and mediterranean plant protection organization. guidelines for the efficacy evaluation of fungicides: *Plasmopara viticola*. *EPPO Bulletin*, 31, 313–317.

Glass, N. L., and Donaldson, G. C. (1995). Development of primer sets designed for use with the PCR to amplify conserved genes from filamentous ascomycetes. *Applied and Environmental Microbiology*, *61*(4), 1323–1330. <https://doi.org/10.1128/aem.61.4.1323-1330.1995>

Udayanga, D., Castlebury, L. A., Rossman, A. Y., and Hyde, K. D. (2014). Species limits in *Diaporthe:* Molecular re-assessment of *D. citri*, *D. cytosporella*, *D. foeniculina* and *D. rudis*. *Persoonia*, *32*, 83–101. <https://doi.org/10.3767/003158514X679984>

Weir, B. S., Johnston, P. R., and Damm, U. (2012). The *Colletotrichum gloeosporioides* species complex. *Studies in Mycology*, *73*(1), 115–180. <https://doi.org/10.3114/sim0011>

White, T. J., Bruns, T. D., Lee, S. B., and Taylor, J. W. (1990). Amplification and direct sequencing of fungal ribosomal RNA genes for phylogenetics. In M. A. Innis, D. H. Gelfand, J. J. Sninsky, & T. J. White (Eds.), *PCR protocols – A guide to methods and applications* (pp. 315–322). New York: Academic Press.
